# Prevalence, Predictors, and Treatment Outcomes of Anemia in Vietnamese School-Age Children With Helicobacter pylori Infection

**DOI:** 10.7759/cureus.69473

**Published:** 2024-09-15

**Authors:** Rang N Nguyen, Nghia Q Bui, Kieu Oanh T Nguyen

**Affiliations:** 1 Pediatrics, Can Tho University of Medicine and Pharmacy, Can Tho, VNM

**Keywords:** anemia, helicobacter pyroli, iron deficiency anemia (ida), iron supplementation, vietnamese children

## Abstract

Background

A growing number of studies suggest that anemia caused by *Helicobacter pylori *(*H. pylori*) infection is prevalent in developing countries. A combination of *H. pylori *eradication and iron supplementation may be effective in treating iron deficiency anemia (IDA) caused by *H. pylori *infection. The aim of this study is to assess the prevalence, risk factors, and treatment outcomes of anemia in Vietnamese school-age children infected with H. pylori

Methods

A cross-sectional study was conducted from April 2023 to April 2024 involving 112 dyspeptic children from 5 to 16 years old who were admitted to Can Tho Children's Hospital, Vietnam. *H. pylori *infection was diagnosed with a positive histopathology combined with a rapid urease test. A bivariate and multivariate logistic regression model was used to identify independent variables associated with anemia in *H. pylori-*infected children.

Results

The prevalence of anemia and IDA (iron deficiency anemia) among *H. pylori*-infected children were 28.6% and 53.1%, respectively. The risk factor for anemia was age group from 11 to 16 years of age (OR: 3.24; CI 95%:1.21- 8.70). Iron supplementation (OR: 0.14; CI 95%:0.03 - 0.66) and periodic deworming (OR: 0.29; CI 95%: 0.10-0.89) were protective factors against anemia. After *H. pyroli *eradication and iron supplementation, all hematological parameters were increased among 32 anemic patients. Hemoglobin increased from 9.9 ±2.1 to 11.9±2.1 g/dL (p<0.001), mean corpuscular hemoglobin increased from 25.1 ± 4.1 to 28.0 ± 3.3 pg (P=0.003), and ferritin (IQR) increased from 16.7 (9.5-45.2) to 27.0 (15.1-47.0) µg/L (p<0.001).

Conclusion

Anemia in Vietnamese school-age children infected with *H. pylori* is prevalent. Taking iron supplements and periodic deworming reduces the risk of anemia. *H. pylori *eradication therapy plus oral iron supplementation will improve iron status in *H. pylori*-infected children.

## Introduction

Infection with *Helicobacter pylori* (*H. pylori*) is one of the most common chronic bacterial infections, affecting approximately half of the world's population. Around 70% of children under the age of 15 in developing countries are infected by this bacterium [1]. In Vietnam, children are highly susceptible to *H. pyroli *infection. A study conducted in Ho Chi Minh City shows that 87.7% of school-age children had *H. pyroli *infection [2]. Among children under the age of 12 with digestive symptoms in North Vietnam, the prevalence reached 92.2% [3]

Children with *H. pylori *infections are typically asymptomatic, but they may develop gastroduodenal ulcers, gastric atrophy, and gastric cancer in the long term [4]. Iron deficiency anemia (IDA) is one of the most concerning extra gastroduodenal disorders associated with *H. pylori *infections [5]. The mechanism of IDA induced by *H. pylori *infection remains unclear. IDA may be caused by blood loss from *H. pylori*-induced mucosal injury and gastroduodenal lesions; impaired iron absorption due to reduced gastric acidity and low ascorbic acid concentration; and competition between *H. pylori *and humans for iron resources [6]

It has been estimated that 35.5% of Vietnamese children under the age of 10 suffer from anemia [7], but no reports have been found of *H. pylori*-infected children suffering from anemia. In a previous study at two Children's hospitals in Ho Chi Minh City, Nguyen et al found that 11.9% of *H. pylori*-infected children had anemia [8]. Risk factors for anemia in Vietnamese children include living in rural areas, poor diet, and intestinal parasite infection [9]. In developing countries, there are several factors that contribute to anemia in school-age children, including food intake practices, female gender, low educational status, and parasitic infections [10]. An *H. pylori *infection may induce anemia in addition to other causes in these children.

There is still controversy surrounding the treatment of IDA caused by *H. pylori *infection. According to some researchers, only *H. pylori *eradication therapy is sufficient without iron supplementation [11,12], but most studies indicate that *H. pylori *eradication therapy combined with iron supplementation is more effective in treating IDA caused by H. pylori infection [13-18],

The aim of this study was to determine the prevalence, risk factors and treatment outcomes of anemia in Vietnamese school-age children infected with *H. pylori*.

## Materials and methods

Study design, setting, and participants

A prospective cross-sectional study was conducted on pediatric patients from 5 to 16 years old who had a gastroduodenal endoscopy because of suspected *H. pylori *infection at Children’s Hospital of Can Tho, Vietnam from April 2023 to April 2024. We selected all available subjects meeting the criteria until the desired sample size was reached.

Sample size

With the assumption of a 95% confidence interval, 6% margin of error, and prevalence of anemia in *H. pylori*-infected children at 11.9% [8], the sample size was found to be 112 subjects.

Inclusion and exclusion criteria

The study included children aged 5-16 with chronic epigastric pain or persistent gastrointestinal disorders. The study excluded children with chronic diseases such as liver cirrhosis, chronic kidney failure or thalassemia, who were taking proton pump inhibitors, bismuth, antibiotics prior to inclusion, or who had concomitant serious diseases, severe gastrointestinal bleeding, or anemia caused by other known causes.

*H. pylori *was detected by a positive urease test (CLOtest) and gastric histological evaluation of biopsy specimens collected through gastroduodenal endoscopy. Anemia was defined according to the World Health Organization (WHO), that is, hemoglobin level of <11.5 g/dL for children under 12 years and <12 g/dL for children equal or above 12 years of age. IDA was defined as anemia plus serum ferritin levels less than 15 µg/dL [19].

All anemia-related parameters including hemoglobin (Hb), mean cell volume (MCV), mean corpuscular hemoglobin (MCH) were performed using SYSMEX-XN 1000 Hematology Analyzer (Sysmex, Kobe, Japan). The serum iron and serum ferritin concentrations were determined using the Beckman Coulter AU 480 (Beckman Coulter, Brea, USA).

We used a pre-designed data collection form to collect socio-demographic data (age, gender, place of residence, mother's education status, mother's occupation, family income) and anemia-related risk factors such as nutrition, iron supplementation, and periodic deworming.

Operational definitions

Undernutrition was defined as a BMI-for-age Z-score below -2SD; an iron-rich diet was classified as yes or no based on the Food Consumption Score Nutritional Quality Analysis (FCS-NQA) [20]; iron supplementation was categorized as yes or no based on regular intake of multivitamins containing iron; periodic deworming every four-to-six months was classified as yes or no; low income was categorized as yes or no according to the Vietnamese government poverty regulations [21]; and residents were classified as either urban or rural.

Ethical issues

Informed consent was obtained from all parents and caregivers at enrolment. This study has been approved by the Science and Technology Board of Can Tho Children's Hospital and the Ethics Committee of Can Tho University of Medicine and Pharmacy (reference number: 949/QĐ-ĐHYDCT). The study was conducted in accordance with the Declaration of Helsinki. The confidentiality of the patients was maintained

Statistical analysis

Statistical Package for Social Sciences (SPSS) version 22.0 (IBM Corp., Armonk, USA) was used to analyze the data. For categorical variables, the values were expressed as numbers and percentages, while for continuous variables, the values were expressed as mean and standard deviation (SD) or median and interquartile range (IQR) as appropriate. A Chi-square test, or Fisher's exact test, was used to analyze categorical data. A Student's t test or a Mann Whitney U test was used to analyze continuous data according to the validity of the normality assumption. A bivariate and multivariate logistic regression model was used to identify independent variables associated with anemia in *H. pylori*-infected children. P-values less than 0.05 were considered statistically significant.

## Results

Socio-demographic characteristics of participants

A total of 112 school-age children with *H. pylori* infection were included in this cross-sectional study. The age of the study participants ranged from 5 to 16 years, with a mean age (±SD) of 10.8 ± 2.7 years. More than half, 58.9% (n = 66), of study participants were female. Around 57.2% (n =64) were urban residents. In terms of the occupational status of mothers (n = 42), 36.5% (n = 42) were private employees, and 18.8% (n = 21) were government employees. A majority of 82.7% of the mothers (n = 93) had completed secondary school. Approximately 67.0% (n = 75) of the children's parents had a middle or high income (Table 1).

**Table 1:** The socio-demographic characteristics of participants

Characteristics	Category	Non-anemia group N=80, n (%)	Anemia group N=32, n (%)	Total, N=112, n (%)
Age (years)	5-10	45 (56.3)	09 (28.1)	54 (48.2)
	11-16	35 (43.8)	23 (71.9)	58 (51.8)
Sex	Male	51 (63.7)	15 (46.9)	46 (41.1)
	Female	29 (36.3)	17 (53.1)	66 (58.9)
Residence	Rural	33 (41.3)	15 (46.9)	48 (42.8)
	Urban	47 (58.8)	17 (53.1)	64 (57.2)
Maternal occupation	Housewife	18 (22.5)	9 (28.1)	27 (24.1)
	Merchant	15 (18.8)	7 (21.9)	22 (19.6)
	Private employee	30 (37.5)	12 (37.5)	42 (37.5)
	Government employee	17 (21.3)	4 (12.5)	21 (18.8)
Maternal education	Primary	12 (15.0)	7 (21.9)	19 (17.0)
	Lower secondary	19 (23.8)	10 (31.3)	29 (25.6)
	Upper secondary	49 (61.3)	15 (46.9)	64 (57.1)
Nutritional status	Undernutrition	17 (21.3)	8 (25.0)	25 (22.3)
	Normal	63 (78.7)	24 (75.0)	87 (77.7)
Family income	Low	22 (27.5)	15 (46.9)	37 (33.0)
	Middle or high	58 (72.5)	17 (53.1)	75 (67.0)

Prevalence of anemia and iron deficiency anemia

The mean hemoglobin (±SD) value of participants was 12.1 ± 1.7g/dL, ranging from 5.3 - 15.3 g/dl. The overall prevalence of anemia was 28.6% (32/112 95% CI: 20.5-37.5). Iron deficiency anemia (IDA) accounted for 53.1% (17/32 95% CI:34.4 -71.9) of anemic patients.

Factors associated with anemia in school-age children with *H. pylori *infection

In a univariate analysis, children with *H. pylori *infection were more likely to be anemic if they were older than 11 years old (Crude Odds Ratio (COR)=3.28, 95% CI: 1.35-7.98) and from a low-income family (COR=2.32 , 95% CI: 0.99-5.44), while iron supplementation (COR=0.24, 95% CI: 0.06-0.86) prevented anemia.

Multivariate analysis showed that children older than 11 years were at greater risk for anemia (Adjusted Odds Ratio (AOR) = 3.24, 95% CI: 1.21 -8.70) , while periodic deworming (AOR= 0.29 , 95% CI: 0.10 - 0.89) and iron supplementation (AOR= 0.14, 95%CI: 0.03 - 0.66) were protective factors against anemia (Table 2).

**Table 2:** Bivariate and multivariable logistic regression analysis of factors associated with anemia in school-age children with H. pylori infection COR: crude odds ratio; AOR: Adjusted Odds Ratio; 95% CI: Confidence Interval

Variables	COR (95% CI)	P-value	AOR (95% CI)	P-value
Sex				
Female	1		1	
Male	1.99 (0.86-4.57)	0.101	2.58 (0.94-7.10)	0.065
Age (years)				
5-10	1		1	
11-16	3.28 (1.35-7.98)	0.009	3.24 (1.21- 8.70)	0.019
Residence				
Rural	1		1	
Urban	0.79 (0.34-1.81)	0.587	0.88 (0.34 - 2.27)	0.795
Undernutrition				
No	1		1	
Yes	1.23 (0.47-3.23)	0.667	1.31 0.43 3.99	0.629
Low income				
No	1		1	
Yes	2.32 (0.99-5.44)	0.049	2.28 (0.82-6.29)	0.111
Iron-rich diet				
No	1		1	
Yes	0.61 (0.23-1.61)	0.321	0.47 (0,15-1.48)	0.203
Iron supplementation				
No	1		1	
Yes	0.24 (0.06-0.86)	0.021	0.14 (0.03 – 0.66)	0.013
Deworming				
No	1		1	
Yes	0.78 (0.33-1.82)	0.577	0.29 (0.10-0.89)	0.031

Treatment outcomes

During this study, 32 anemic patients were treated with *H. pylori *eradication using the triple standard regimen of proton pump inhibitors, amoxicillin and metronidazole (or clarithromycin) in 2 weeks, combined with elemental iron supplementation at 3 mg/kg body weight/day in 1 month. After treatment, mean Hb (±SD) increased from 9.9± 2.1 to 11.9± 2.1 g/dL (P=0.002). MCH (±SD) increased from 25.1± 4.1 to 28.1± 3.3 pg (P=0.003), median (IQR) ferritin increased from 16.7 (9.5-45.2) to 27.0 (15.1-47.0) µg/L (P=0.018), and median (IQR) serum iron increased from 9.9 (5.9-15.9) to 18.4 (12.4-20.3) µmol/L (P=0.025) (Table 3).

**Table 3:** Changes in hematological parameters after 1-month treatment MCV: Mean Corpuscular Volume; MCH: Mean Corpuscular Hemoglobin * Student’s T test ** Wilcoxon Signed Ranks Test

Hematological parameters	Reference range	At baseline (N=32)	After 1 month (N=32)	P-value
Hemoglobin, mean±SD	11.5-15.5 g/dL	9.9 ± 2.1	11.9 ± 2.1	0.002*
MCV, mean±SD	73-95 fL	78.0 ± 12.4	82.3 ± 7.6	0.151*
MCH, mean±SD	24-33 pg	25.1 ± 4.1	28.1 ± 3.3	0.003*
Ferritin, median (IQR)	7.0-140 µg/L	16.7 (9.5-45.2)	27.0 (15.1-47.0)	< 0.001**
Iron, median (IQR)	3-25 µmol/L	9.9 (5.9-15.9)	18.4 (12.4-20.3)	0.001**

## Discussion

In this study, we found that the prevalence of anemia was 28.6% (95% CI: 20.5-37.5) and 53.1% (95% CI: 34.4 -71.9) had IDA among school-age children with *H. pylori *infection. For children aged 5 to 10, iron supplementation and periodic deworming tended to be protective factors against anemia. Hematological parameters associated with anemia (hemoglobin, mean corpuscular hemoglobin, serum ferritin, and serum iron) increased after 1-month treatment compared to baseline (Table 3).

The prevalence of anemia in school-age children with *H. pyroli *infection in our study was higher than the previous prevalence of anemia (11.9%) in *H. pylori*-infected children who underwent endoscopy at the Children's Hospital of Ho Chi Minh City, Vietnam [9 ] and also higher than those found in other studies in China, Iran, and Romania ranging from 6.2 to 8.8% [22-24], but lower than studies in *H. pylori*-infected children in Turkey (57%) [25].

In this study, prevalence of anemia (28.6%) caused by *H. pylori *infection is lower than the prevalence of anemia (35.5%) for all causes in a previous Vietnamese meta-analysis. [7], suggesting the *H. pylori *infection may not have been the only factor of anemia in our population. 

Iron supplementation and deworming reduced the risk of anemia in our study population. Therefore, anemic children may be caused by parasitic infection itself or by co-infection with *H. pyroli*. Previous research studies have found that co-infection with *H. pylori *and other intestinal parasites ranges from 23% to 44.3% in different populations [26, 27]. Additionally, school-age children aged 11-16 had a higher risk of anemia than those under 11 years of age. This may be due to accelerated requirements for iron, poor dietary intake of iron, parasite infestations, and menarche in girls at this age. This is consistent with the WHO report in Southeast Asia that the age group 12-15 years old, especially girls are at the highest risk of anemic prevalence of more than 25%. [28]. Therefore, the risk factors associated with anemia in children with *H. pylori *in this study are no different from those due to other causes of anemia previously recorded in Vietnam, including living in the countryside, intestinal parasite infection, and a diet lacking in meat and fish [9].

The evidence for iron deficiency anemia caused by *H. pylori *infection is currently lacking [29]. Therefore, iron supplementation and *H. pylori *eradication are necessary in *H. pylori*-infected children. In our pediatric population, all anemia-related parameters (Hb, MCH, serum ferritin, and serum iron) improved after one month of treatment. Our study shows similar results to those of previous studies on *H. pylori *infection-induced anemia in children treated with anti-*H. pylori *medication and iron supplementation [13, 16-18, 30].

Research by Duque et al. on Mexican children with *H. pylori *infection found that children with *H. pylori *eradication and ferrous sulfate supplementation had an increase in Hb mean concentration of 0.47g/dL (95% confidence interval, 0.01, 0.93), while those with *H. pylori *eradication without iron supplementation had an increase of 0.37g/dL (95% CI, 0.02, 0.75) [13]. In a randomized controlled study of children infected with *H. pylori *and anemia in India, Valiyaveettil et al. found that children receiving anti-*H. pylori *treatment plus oral ferrous sulfate for three months had higher levels of Hb and ferritin than children receiving iron supplementation without anti-*H. pylori *treatment (Hb 3.6 versus 1.1 g/dL; ferritin 55.5 versus 19 mcg/dL) [18]. A study by Sarker et al. on *H. pylori*-infected Bangladeshi children reported that Hb and ferritin increased more in the anti-*H. pylori *plus three-month iron supplementation group (Hb increased 16±12 g/L, ferritin increased 53 ±11 mmol/L) than in the anti-*H. pylori *without iron supplementation group (Hb increased 7±10 g/L, ferritin increased 10±2 mmol/L) [17]. A meta-analysis that included 16 randomized control trials found that oral iron supplementation combined with *H. pylori *eradication therapy increased Hb, serum iron, and ferritin levels more than iron supplementation alone (standardized mean difference, Hb 1.48; 95% CI, 0.96, 2.00; p < 0.001; serum iron 1.15; 95% CI, 0.87, 1.43; p < 0.001; serum ferritin 1.84; 95% CI, 1.20, 2.48; p < 0.001, respectively)[30]

A strength of this study was that all *H. pylori*-positive children were identified by histological examination and a positive urease test. However, the study had some limitations: First, a small sample size of 112 poses challenges for generalization. Second, the effect of parasites on iron absorption was not assessed due to the lack of stool tests. Third, IDA can be misdiagnosed due to hyperferritinemia caused by inflammation in some patients. Lastly, the short post-treatment follow-up period.

## Conclusions

Anemia in Vietnamese school-age children infected with *H. pylori *is prevalent. *H. pylori*-induced anemia has no different risk than anemia caused by other causes. Taking iron supplements and deworming regularly reduces the risk of anemia. A combination of *H. pylori *eradication therapy and oral iron supplementation will improve iron status in school-age children with *H. pylori *infection.
